# Extracting scientific articles from a large digital archive: BioStor and the Biodiversity Heritage Library

**DOI:** 10.1186/1471-2105-12-187

**Published:** 2011-05-23

**Authors:** Roderic DM Page

**Affiliations:** 1 Institute of Biodiversity, Animal Health and Comparative Medicine, College of Medical, Veterinary and Life Sciences, Graham Kerr Building, University of Glasgow, Glasgow G12 8QQ, UK

## Abstract

**Background:**

The Biodiversity Heritage Library (BHL) is a large digital archive of legacy biological literature, comprising over 31 million pages scanned from books, monographs, and journals. During the digitisation process basic metadata about the scanned items is recorded, but not article-level metadata. Given that the article is the standard unit of citation, this makes it difficult to locate cited literature in BHL. Adding the ability to easily find articles in BHL would greatly enhance the value of the archive.

**Description:**

A service was developed to locate articles in BHL based on matching article metadata to BHL metadata using approximate string matching, regular expressions, and string alignment. This article locating service is exposed as a standard OpenURL resolver on the BioStor web site http://biostor.org/openurl/. This resolver can be used on the web, or called by bibliographic tools that support OpenURL.

**Conclusions:**

BioStor provides tools for extracting, annotating, and visualising articles from the Biodiversity Heritage Library. BioStor is available from http://biostor.org/.

## Background

In July 2010 Lambert et al. [1] published a paper in *Nature *that described an extinct sperm whale possessing the biggest bite of any tetrapod known. They named this formidable predator *Leviathan melvillei*, the genus name *Leviathan *being derived from the Hebrew 'Livyatan', the species name honouring Herman Melville (author of Moby Dick [2]). As appropriate as this name was, it quickly ran foul of the rules of zoological nomenclature [3] because *Leviathan *had been used 169 years previously for an extinct species of mammoth [4]. Although the name *Leviathan *Koch [4] had lapsed into obscurity (as a synonym of *Mammut *Blummenbach) its existence meant the newly discovered whale had to be renamed, which it duly was a month after the original publication [5].

The fate of Lambert et al.'s *Leviathan *illustrates a significant challenge facing researchers finding and naming new species - the discoverability of existing names. In the absence of a global register of all taxonomic names that have ever been published, a researcher about to publish a new name may struggle to establish that that it has not already been used. Zoological nomenclature dates from 1758, botanical nomenclature from 1753, hence a comprehensive list of taxonomic names must survey some 250 years of literature [6], much of which is obscure and may not exist in digital form. Digitising this legacy literature is the goal of the Biodiversity Heritage Library (BHL) [7,8], a consortium of natural history museum libraries, botanic libraries, and research institutions. The bulk of this digitisation is carried out by the Internet Archive [9], which scans books (broadly defined to include bound issues of journals), creating a set of electronic files for each scanned item, which includes images of individual pages, and text extracted from those pages using Optical Character Recognition (OCR). BHL takes these files (together with the output from the scanning projects of individual BHL members), indexes them by bibliographic metadata and taxonomic names, and makes the content available on its web site [7] (both as web pages and web services). Although the bulk of BHL's scanning activities focus on pre-1923 content that is out of copyright, it has not inconsiderable post-1923 content contributed by its member institutions, notably publications by various natural history museums.

The inability to easily locate articles in BHL is a substantial obstacle to integrating this legacy biodiversity literature into mainstream scientific publishing. The goal of BioStor is to provide tools to locate and extract articles from the BHL archive. BioStor differs from search engines such as PubMed [10] and Google Scholar [11], which support free-form queries such as "what articles have been published on this topic?", or "what papers has this author published?" BioStor addresses a different question, namely "does this article exist in the BHL archive?" It is a tool to find out whether a specific article exists in the archive, as opposed to finding what articles exist on a particular topic.

### Locating articles in BHL

**Figure 1:**
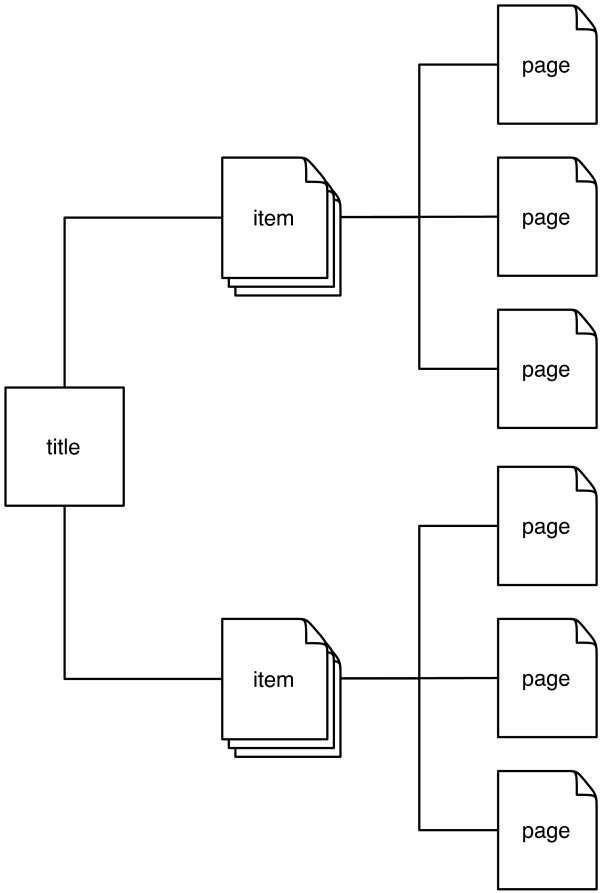
**Simplified model of Biodiversity Heritage Library content**. Each scanned item comprises one or more page images. Items are grouped together into titles.

The BHL archive comprises "items" corresponding to physical objects which are scanned. Items are grouped together into "titles". A single volume book corresponds to a single title and item, whereas a multi-volume work, such as a journal, will comprise several items grouped under the same title (Figure 1). Noticeably absent from the BHL model is the standard unit of scientific citation, the article.

For most modern articles the triple of journal name, volume, and starting page is sufficient to uniquely identify an article [12], and tools such as CrossRef's OpenURL resolver [13] can take this this triple and discover whether a Digital Object Identifier (DOI) [14] exists for a that article. Publishers make use of this tool to map the literature cited in a manuscript to the corresponding DOI. In an ideal world the BHL model of (title, item, page) (Figure 1) would map exactly to (journal, volume, page), such that an individual journal would correspond to a title in BHL, and each volume of that journal was a separate item. Given that BHL stores page numbers for each scanned page [8], locating articles would then be trivial and linking to BHL content could be readily integrated into existing publication processes, as well as bibliographic management tools that make use of CrossRef's services to augment user-provided metadata (e.g., Mendeley [15]).

Unfortunately, the actual mapping between articles and BHL content is often rather more complicated. Large articles (e.g., monographs) may be treated as separate "titles" (effectively as if they were books), rather than parts of the same title. A contributing library may have bound several volumes of a journal together, such that a single "item" may comprise multiple volumes. Volume numbers themselves may not be unique within a journal. *The Annals and Magazine of Natural History *(ISSN 0374-5481), published from 1828 until 1967 (being succeeded by the *Journal of Natural History*, ISSN 0022-2933), is divided into 13 "series", each series numbering its volumes from one onwards. Hence, "volume 1" of *Annals and Magazine of Natural History *may refer to any one of 13 volumes spanning 138 years [16]. Journals also differ in whether pagination is unique within a volume, or within parts of a volume. For example, in the journal *Arkiv för Zoologi *(ISSN 0004-2110) each article starts on page 1, so that the triple (*Arkiv för Zoologi*, 13, 1) may refer to [17,18], or any of 23 other articles in volume 13 of that journal.

Discovering articles also assumes that the pagination in BHL is complete and correct, and that one side of a sheet of paper corresponds to a "page". BHL records the page number of regular pages, but not pages that are classified as special in some way, such as title pages, or tables of contents. For example, page 1 in Lynch et al. [19] is recorded in BHL as being the title page without any number, which will frustrate efforts to locate this article by starting page alone.

**Figure 2:**
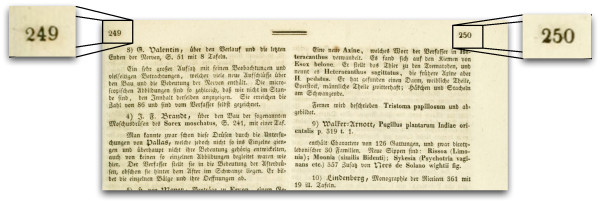
**Physical page with two page numbers**. Example of a physical page in the journal *Isis von Oken *with two columns, each of which as its own page number (249 and 250, respectively)

While the triple (journal, volume, starting page) is usually sufficient - subject to the caveats above - to locate the start of an article, we want to recover all the pages in the article, hence we need both the starting and ending pages. Ideally we could then extract the corresponding set of page images from BHL and join them together to form an article. However, it is not uncommon for older articles to have discontinuous physical pagination, for example by having plates inserted between pages in the text. In some publications, such as *Isis von Oken*, the text on a page forms two columns, each with its own page number (Figure 2), hence one physical page need not equate to a bibliographic page.

### Metadata matters

Given that locating articles in a archive of legacy literature such as BHL is a non-trivial task, it is worth considering why such an undertaking is worthwhile, beyond integrating BHL with existing citation practices. Indeed, one could argue that, given that the OCR text for BHL content has been indexed by taxonomic name, the need for indexing by article has been greatly reduced - the user could simply search by taxonomic name and find the content they require. This would be sufficient for many users, especially if we were con fident that BHL had correctly indexed all the taxonomic names contained in the pages it has scanned. However, OCR errors mean that a significant fraction of names will be missed [20]. An obvious approach to discovering these missing names would be to take existing databases of taxonomic names and publications and search for those publications in BHL.

**Figure 3:**
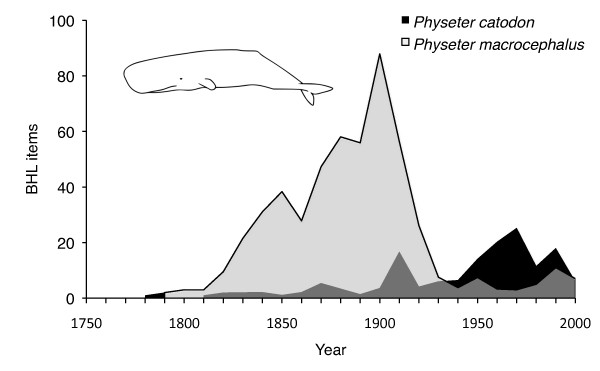
**Usage of two names for the sperm whale over time**. Approximate distribution over time of two alternative names for the sperm whale (*Physeter catodon *and *Physeter macrocephalus*) in items scanned by the Biodiversity Heritage Library. Date of publication was extracted from the StartYear and EndYear fields of the Title table (see Fig. 4) using regular expressions.

Metadata also provides ways for clients to aggregate and filter search results. The Encylopedia of Life [21] incorporates search results from BHL in its taxon pages, but the user has no obvious means of discovering whether the results are from the same article or not, nor can they order the results by date. As an example of one way the display of search results can be improved by sorting, consider the dispute concerning the correct scientific name for the sperm whale, which is debated in both the scientific literature [22-24] and, more vociferously, Wikipedia [25]. Being able to extract basic metadata from BHL would enable us to visualise the relative popularity of the two alternatives, *Physeter catodon *and *Physeter macrocephalus*, over time (Figure 3). With the obvious caveat that the literature in BHL is a biased sample of the taxonomic literature, it is clear that *Physeter macrocephalus *is the more commonly used name, but its usage peaked around the start of the twentieth century. By the 1950, the sperm whale was more commonly refered to as *Physeter catodon*. Navigating BHL content by date may help the user discover why the relative usage frequency of these two names changed in the previous century.

## Construction and content

**Figure 4:**
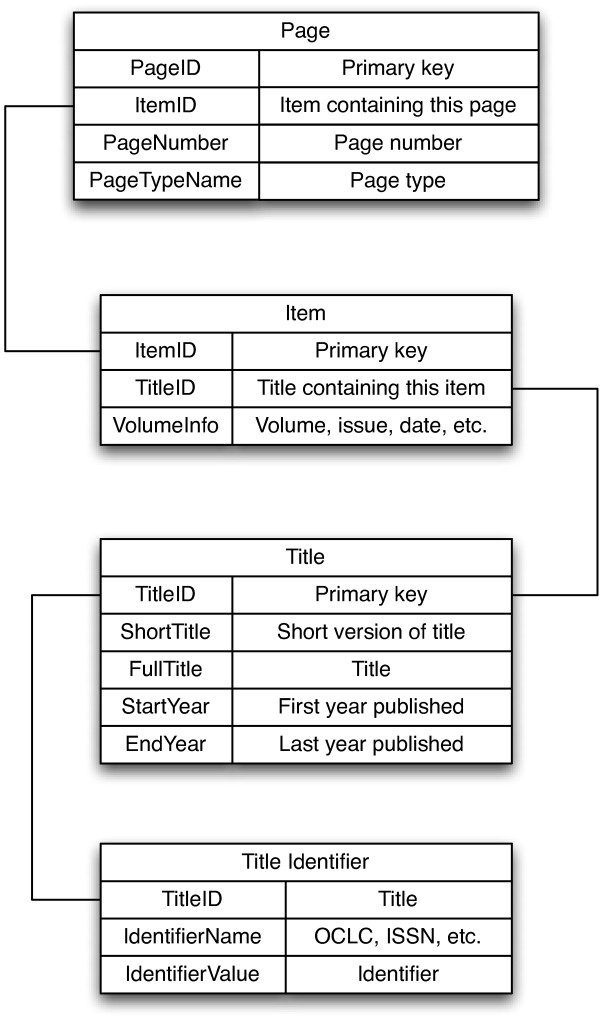
**Simplified BHL schema**. Simplified database schema for the core tables in the Biodiversity Heritage Library. The fields referred to in the text are shown, together with a brief explanation of their contents.

A local copy of the core BHL tables (Figure 4) was created in MySQL using the data dump provided by BHL http://www.biodiversitylibrary.org/data/data.zip. Page images and OCR text for individual pages are retrieved as needed using the BHL API and cached locally (together with a thumbnail of the page image).

### Locating an article

**Figure 5:**
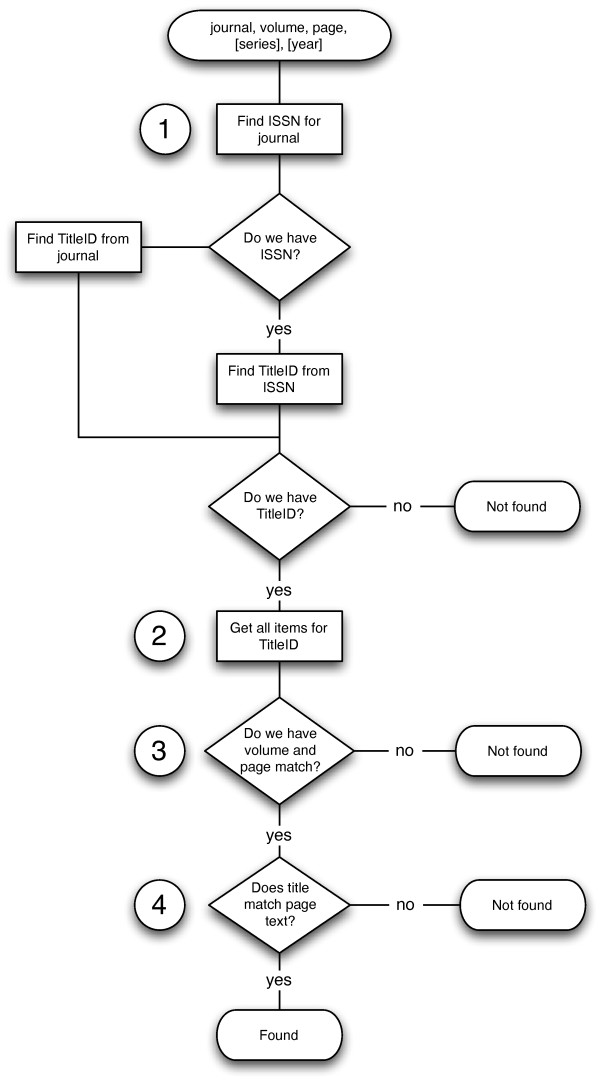
**Flow chart of algorithm for finding an article in BHL**. Steps 1-4 are explained in the text.

BioStor provides an OpenURL [26] resolver service to locate articles in BHL. At a minimum the resolver requires the journal name, volume, and starting page of the article being searched for. It may also make use of journal series and date, if these are provided. This service first checks whether the article already exists in the BioStor database. If the article is not found, the algorithm outlined in Figure 5 is used to search for the article in BHL.

#### Step 1 - Finding the journal

The first step is to determine whether BHL includes the journal containing the article. BioStor uses a service provided by bioGUID [27,28] to find the ISSN [29] for the journal. If the bioGUID service returns an ISSN, the algorithm looks up the ISSN in the Title Identifier table (Figure 1) and retrieves the corresponding BHL TitleID. If the bioGUID service doesn't return a ISSN the algorithm attempts to find the journal title in the ShortTitle field in the Title table using approximate string matching. If it fails to find the title it then searches the VolumeInfo field in the Item table - for some journals (e.g., *Fieldiana Zoology*, ISSN 0015-0754) the journal title is stored in that field. If at this point we can't find the journal we exit.

#### Step 2 - Finding scanned items for the journal

Ideally each journal corresponds to a single BHL title, but in some cases the same journal may be represented by more than one BHL title, and hence have more than one TitleID. Step 2 uses a hard-coded table of such cases to ensure that all items for a given journal are considered by Step 3.

#### Step 3 - Finding the volume and page

Ideally the VolumeInfo field in the Item table would contain just the volume number, however all manner of free-form text may be found there. The volume may be recorded as simple numbers or as strings, sometimes indicating volume, page or date ranges, notes on completeness of the volume, or other comments (e.g., "Index"). Metadata may also be in a variety of languages, such that the field may refer to "Volume", "Band", or "Tome". Nor is metadata always recorded consistently within a journal, for example the VolumeInfo field for scanned items belonging to the journal *Proceedings of the Zoological Society of London *contains strings such as:

• Part 1- Part 4 (1833-38)

• 1856

• 1901, v. 1 (Jan.-Apr.)

• Jan-Apr 1906

• 1912 v. 2

• 1923, pt. 1-2 (pp. 1-481)

BioStor uses a set of ad-hoc regular expressions to extract volume (and other information where present, such series, issue, and date) information from the VolumeInfo field. If no match to the target volume is found the algorithm exits.

#### Step 4 - Checking the match

**Figure 6:**
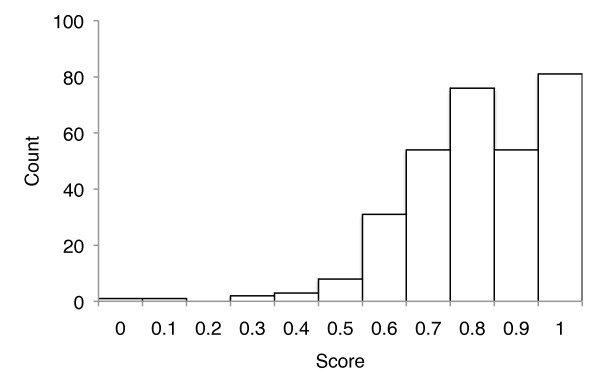
**Alignment scores for Annals and Magazine of Natural History**. Frequency distribution of scores for Smith-Waterman alignment between article title and OCR text for 314 articles from *Annals and Magazine of Natural History *in the Biodiversity Heritage Library.

At this stage in the algorithm we will have one or more candidates for the first page in the article. Multiple candidates may occur because the article has been scanned by more than one BHL contributor, or because there may be more than one article with the same metadata (see examples of *Annals and Magazine of Natural History *and *Arkiv för Zoologi *discussed above). Some of these matches can be filtered by series or date, if the user has supplied that information. For each remaining match we take the OCR text for the first page in the candidate and compare it to the article title by computing a local alignment between words in the page and word in the title using the Smith-Waterman [30] algorithm. Each pair of words that match exactly are scored +2, mismatches, deletions, and insertions are all scored -1. The score for the alignment is normalised by the match score × the number of words in the title, so that a perfect match has a score of 1. As an illustration, Figure 6 shows the distribution of alignment scores for the *Annals and Magazine of Natural History*. Most articles in this journal have a score > 0.5, however some articles have very low scores due to poor OCR quality. For example, for the article "Preliminary notice of the Schizopoda collected by H. M.S. Discovery in the Antarctic region" [31] the corresponding OCR text is "Preltiniiiari/Xutice of I he Sc/ti:oj/0(/a collcxted hy 11. M.S. 'Dixcovenj' in the Antarctic Rec/io".

### Storing articles

**Figure 7:**
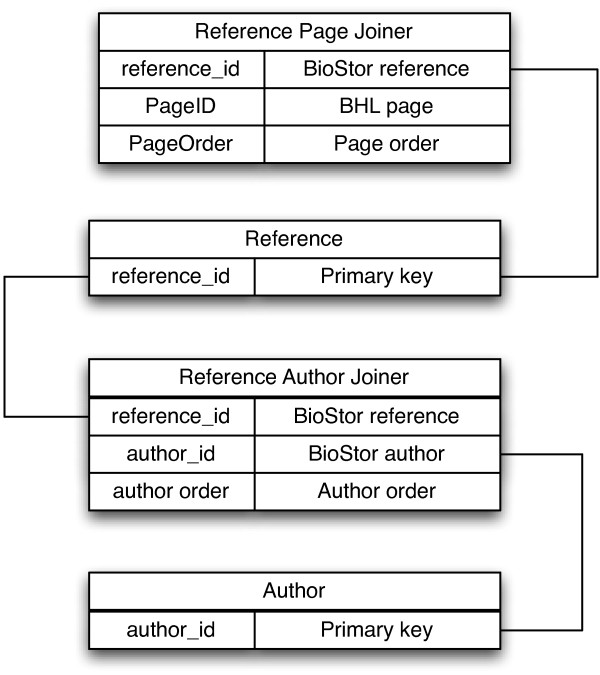
**Simplified BioStor database schema**. Simplified database schema for the core tables in the BioStor database.

Articles extracted from BHL are stored in the same MySQL database that stores the BHL tables, using a simple schema comprising a table for article bibliographic metadata, a table for authors, and a table that joins the authors to the individual articles they've authored. A further table joins the article to the BHL Page table (Figure 7).

## Utility and Discussion

The BioStor database is available at http://biostor.org/. It features an OpenURL resolver, and can display individual articles, lists of publications by author, by taxonomic name, and by journal. At the time of writing the database contains 26,784 articles extracted from BHL.

### OpenURL resolver

**Figure 8:**
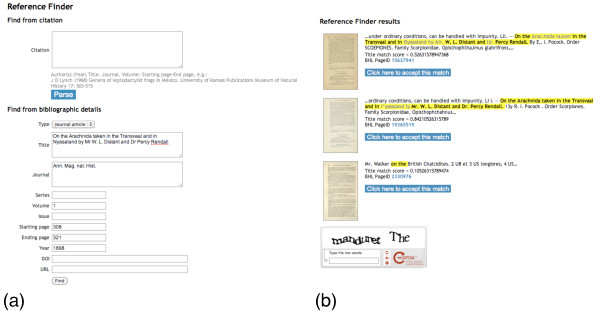
**BioStor OpenURL resolver**. (a) Example of using the web interface to the OpenURL resolver. The user has entered bibliographic details for the reference "On the Arachnida taken in the Transvaal and in Nyasaland by Mr W. L. Distant and Dr Percy Rendall" [53]. (b) The resolver has found three possible matches in the Biodiversity Heritage Library. For each match the best alignment between the article title and the OCR text is highlighted in yellow. The user can then chose which match will be stored in BioStor.

BioStor provides an OpenURL resolver at http://bioguid.info/openurl/. If accessed using a web browser the user is presented with a form where they can enter the bibliographic details of an article individually (Figure 8a), or paste in a full citation and have BioStor attempt to parse it. BioStor's article parser uses regular expressions and is limited to simple citations of the form <author(s)> <(Year)> <article title>. <journal>. <volume>: <starting page>-<end page>. If the article is already in the BioStor database the article will be displayed, if not BioStor attempts to locate the article in BHL. If it finds potential matches, these are displayed to the user (Figure 8b). For each match the page displays the score based on Smith-Waterman alignment between the page OCR text and the article title. In the example shown in Figure 8b, there are three potential matches, two of which have high scores (they are duplicates resulting from two BHL contributors having scanned the same journal). A thumbnail of the first page in each possible match is shown, the user can click on this to view a larger version of the page if they wish to inspect the match more closely. If they are happy that one of the matches is indeed the article they were looking for, the user can fill in the reCAPTHCA test [32,33] and click on the corresponding button. BioStor will then retrieve the remaining page images and OCR text from BHL, store the article in its database, then display it to the user.

Cutting and pasting bibliographic details into web forms is tedious, so the web interface to the OpenURL resolver is intended for casual use only. Instead, it is envisaged that users will interact with the OpenURL resolver using one of the bibliographic tools that supports the protocol, such as EndNote [34] and Zotero [35], or a web browser that supports OpenURL ContextObject in SPAN (COinS) [36], such as Firefox with the OpenURL Referrer add on [37]. For example, the following OpenURL corresponds to the web form shown in Figure 8a (with line breaks added for clarity):

http://biostor.org/openurl

?genre=article

&atitle=On the Arachnida taken in the Transvaal and in Nyasaland by Mr W. L. Distant and Dr Percy

Rendall

&title=Ann. Mag. nat. Hist.

&volume = 1

&spage = 308

&epage = 321

&date = 1898

Appending "&format=json" to the OpenURL returns the result in Javascript Object Notation (JSON), hence the service can be used as an API by other developers.

### Retrieval performance

The ability of BioStor to find articles in BHL depends on several factors. An obvious reason BioStor may fail to find an article is that it simply has not been scanned by BHL. Alternatively, it may have been scanned by BHL but not yet added to the local copy of BHL used by BioStor. Even if an article exists in BHL, BioStor may fail to find it if the metadata describing the item that contains the article doesn't conform to one of the regular expressions BioStor uses to interpret the VolumeInfo field in the Item table. Because BioStor evaluates the quality of a match by comparing the title of the target article with the OCR text (Figure 6), OCR errors may result in the match being deemed too poor to be correct. If the metadata for the target article contains significant errors, such as incorrect pagination, then BioStor may also fail to find an article.

#### Retrieval of articles in the journal Tijdschrift voor Entomologie

To provide a benchmark for BioStor's performance I used an EndNote database of 2330 articles from the journal *Tijdschrift voor Entomologie *spanning the years 1858 to 1999, inclusive, assembled by E. J. van Nieukerken as part of a complete index of the journal [38]. Almost all volumes of *Tijdschrift voor Entomologie *for this period have been scanned by BHL, so ideally BioStor should recover most, if not all of these articles from this journal. This database chosen because of the quality of the bibliographic metadata, and the fact it spanned some 150 years, during which time the typeface and layout of the journal changed significantly.

The EndNote file for *Tijdschrift voor Entomologie *was converted into a Research Information Systems (RIS) format file, which was then parsed by a script which extracted each article, constructed an OpenURL query, and forwarded it to BioStor, which returned a response in JSON format. The script scored recorded whether a match for article was found, ignoring matches with an alignment score of less than 0.5. As part of the output the script created web pages displaying details of each putative match including a thumbnail image of the first page of the article, making it possible to quickly evaluate whether the match was correct. The database, scripts, and HTML output are available from http://biostor.org/ms/.

**Figure 9:**
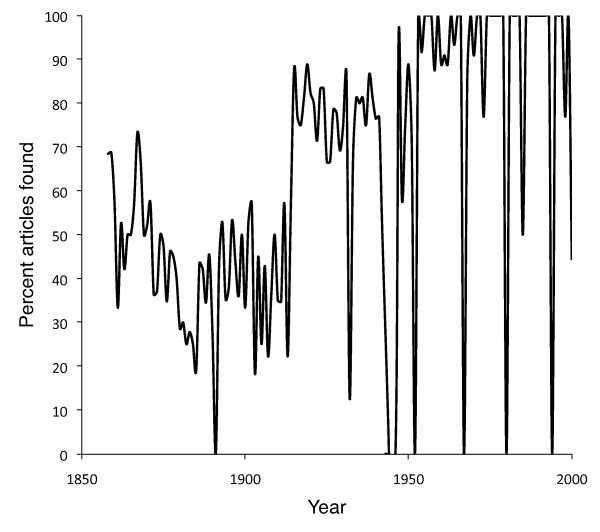
**Success in locating articles from the journal Tijdschrift voor Entomologie**. Percentage of articles in the journal *Tijdschrift voor Entomologie *for the years 1858-1999 that BioStor found in the Biodiversity Heritage Library (BHL). 0% values represent volumes of *Tijdschrift voor Entomologie *that have not been scanned by BHL.

Of the 2330 articles in the database, 94 articles are in volumes not presently available in BHL, and 224 articles have pages labelled with Roman numerals which weren't recorded by BHL. This left 2012 articles in the BHL archive, of which BioStor found matches for 1429 (71%), doing noticeably better for articles published after 1950 (Figure 9). Only fifteen matches (1%) were found to be incorrect, in each case due to pagination errors in the corresponding scanned items in BHL (typically the pagination recorded by BHL was offset from the correct pagination by 2-3 pages).

*Tijdschrift voor Entomologie *is just one of the journals scanned by BHL, and it would be desirable to evaluate BioStor's performance across a range of journals. However, at present evaluation is hampered by the lack of freely available, comprehensive bibliographic databases for taxonomic journals.

### Displaying articles

**Figure 10:**
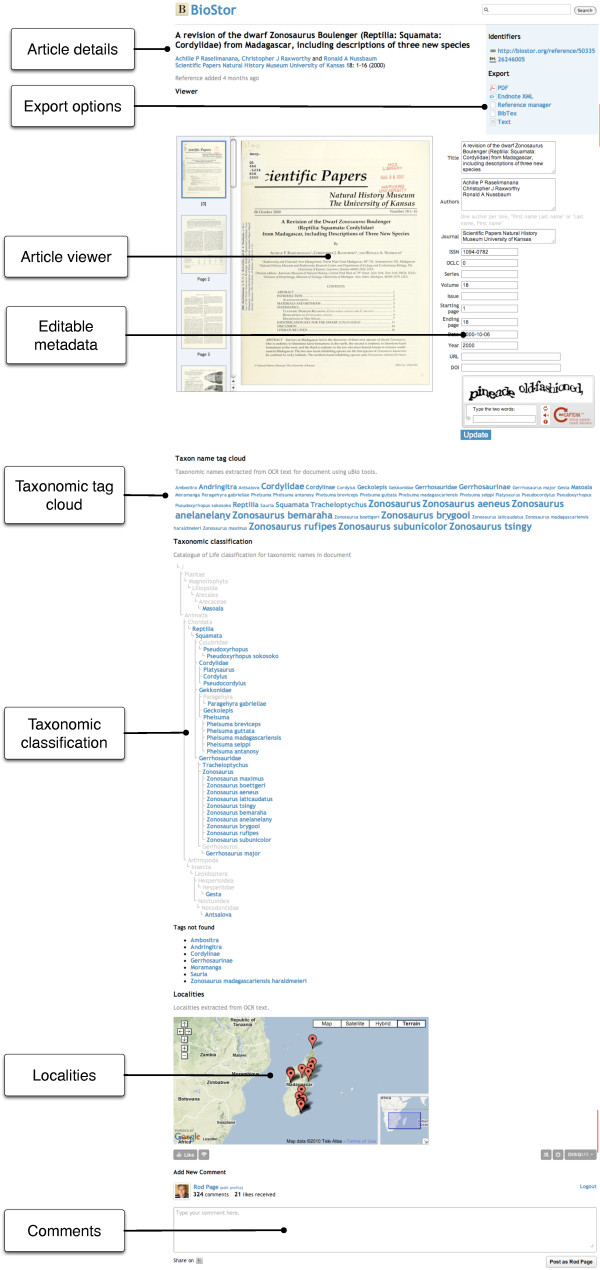
**Example of page displaying an article in BioStor**. The article being displayed is [39].

Articles found by the OpenURL resolver are stored in the BioStor database, and given a unique URL of http://biostor.org/reference/n where *n *is a unique integer. Figure 10 shows an article [39] being displayed in BioStor. A simple Javascript-based viewer displays a single page as a image, with thumbnails of the all the pages in the article shown in a scrolling list. To minimise the time the article page takes to load the thumbnails are only loaded when visible using a delayed Javascript image loader [40]. The user can navigate through the article by clicking on the thumbnail for a given page. To smooth the transition between individual pages, when the user clicks on the thumbnail for a new page the thumbnail is displayed in place of the full page image while that page image loads. When the page image has loaded the low resolution thumbnail (which will appear fuzzy to the user) is replaced by the higher resolution image, giving the user the sensation that the page has come into focus.

The metadata (such as title, authors, journal name, etc.) can all be edited by the user. These edits will be saved if the user passes a reCAPTHCA test. The metadata can be retrieved in standard formats such as Reference Manager (RIS), Endnote XML, and BibTeX. The web page also contains bibliographic metadata embedded using the Context Object in Span (COinS) technique [36], and <meta> tags using the Dublin Core [41] and Google Scholar [11] vocabularies. The article itself can also be downloaded as a PDF file, with bibliographic metadata embedded using Adobe's Extensible Metadata Platform (XMP) [42]. Desktop bibliographic software that can read XMP, such as Mendeley [15,43] and Papers [44], can extract this metadata so that the user need not manually re-enter bibliographic details for the paper.

The article page also displays the taxonomic and, where possible, geographic scope of the article. Taxonomic scope is represented by a tag cloud of the taxonomic names that BHL has found in the OCR text for the article, and by a taxonomic classification of those names based on the 2008 edition of the Catalogue of Life [45]. When an article is added to the BioStor database the OCR text is searched for strings that represent latitude and longitude values for point locations. Any points found are displayed on a Google Map.

### Displaying authors

BioStor displays a summary page for each author in the database. To mitigate the problem of an author having more than one spelling of their name, BioStor clusters names using a web service provided by bioGUID [27], which implements Feitelson's [46] weighted clique algorithm for finding equivalent names. The summary page aggregates publications and coauthorships across this set of names. The page uses Exhibit [47] to create a faceted browser, enabling the user to browse an author's publications by date, journal, and coauthors.

### Displaying journals

**Figure 11:**
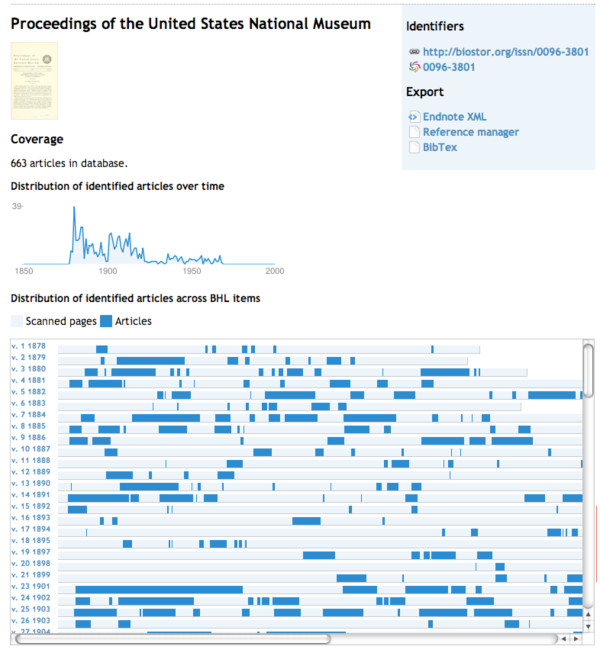
**Summary of coverage of the journal Proceedings of the United States National Museum in BioStor**. Dark blue bars represent pages that have been assigned to an article in BioStor. A sparkline depicts the distribution of these articles over time.

By default BioStor uses the ISSN to identify journals. Where a ISSN isn't available BioStor uses an OCLC number from the WorldCat service [48]. A user can see all the articles for a given journal by appending the journal's ISSN to the URL http://biostor.org/issn/ (or OCLC to the URL http://biostor.org/oclc/). The resulting web page lists the articles for that journal, as well as a graphical representation of how many articles for that journal have been located in BHL. Figure 11 shows the coverage of the journal *Proceedings of the United States National Museum *(ISSN 0096-3801), published from 1878 to 1968.

### Displaying taxonomic names

If the user clicks on a name in the taxonomic tag cloud (Figure 10), or appends a taxonomic name (or uBio NameBankID [49]) to the URL http://bioguid.org/name/ for a name that has been taxonomically indexed by BHL, BioStor displays a web page listing the articles in BioStor that contain that name. The page also displays a sparkline showing the distribution of that name over time in the local copy of BHL, and lists taxonomic synonyms of the name according to the 2008 edition of the Catalogue of Life [45].

### Searching and browsing

**Figure 12:**
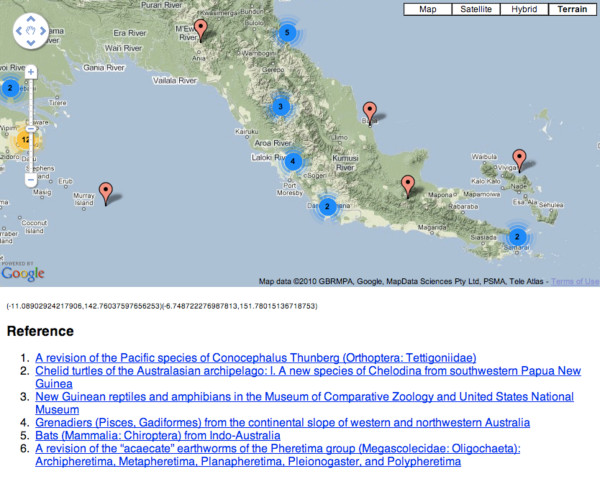
**Browsing BioStor content geographically using Google Maps**. Listed below the map are the articles in the BioStor database with localities contained within the geographic area being displayed in the map.

BioStor supports rudimentary full text search of author names and article titles. It also provides an interactive way to browse articles geographically using Google Maps http://biostor.org/maps/ (Figure 12). When the user pans or zooms the map the web page displays the set of articles (up to a limit of 20) whose OCR text includes (latitude, longitude) pairs contained within the current bounds of the map.

### Future directions

BioStor locates articles by matching existing bibliographies to BHL content, hence it relies on external sources of metadata to find articles. Typically these are bibliographies assembled by individual taxonomists for particular taxonomic groups, or lists of articles published in a single journal. An alternative approach would be to extract articles directly from the archive. Lu et al. [50] used feature extraction and a mixture of rule-based and machine-learning techniques to extract metadata from BHL OCR text, recovering between 66% to 94% of articles in selection of three journals. The set of articles in BioStor could be used as a training data set to help further develop these methods. Another approach to article extraction is crowd sourcing, where the task of identifying articles would be devolved to users. Ultimately, crowd sourcing could become important in cleaning metadata, but it may prove challenging to engage users in creating metadata from scratch.

The BHL archive has extracted taxonomic names from the OCR text, and BioStor looks for geographic localities encoded as latitude and longitude pairs. We could make more extensive use of the OCR text, for example by using autonomous citation indexing [51] to extract citations from the literature cited section of each article. These citations could in turn be feed into the BioStor OpenURL resolver to attempt to locate them in BHL. The combination of variable citation styles and OCR errors means that the same reference may have be represented by several different citations, requiring tools for cleaning and merging citation data (e.g., [52]).

BioStor is built as a service on the top of a copy of data from BHL, and creates a local bibliographic database of articles. One future direction would be to integrate this data with BHL itself. BHL has an OpenURL resolver http://www.biodiversitylibrary.org/openurlhelp.aspx that primarily supports books rather than articles. Adding metadata from BioStor could enhance the BHL OpenURL service, and provide the biodiversity community with a single source for BHL-derived content. BioStor content could also be added to other bibliographic databases, in particular Mendeley [15,43]. Mendeley is developing an API for storing and retrieving documents and associated metadata, hence it might be possible to devolve the storing of basic bibliographic metadata to Mendeley, BioStor then becoming simply an OpenURL resolver.

## Conclusions

The 31 million scanned pages made available by the Biodiversity Heritage Library (BHL) represents a substantial resource of biological literature. BioStor provides an OpenURL resolver to locate articles in this archive. Each article extracted from BHL is given a unique URL, corresponding to a web page that displays the article pages, and information about the taxonomic names and geographic localities mentioned in the article. BioStor is available at http://biostor.org/.

## Availability and requirements

• **Project Name: **BioStor

• **Project Home Page: **http://biostor.org/. Source code is available from http://code.google.com/p/bioguid/source/browse/#svn/trunk/biostor.

• **Operating System: **The BioStor web site is usable with any modern web browser. The source code can be easily installed on a Mac OS X, Linux server. It has not been tested on a Windows machine.

• **Programming Language: **PHP

• **Other Requirements: **Web server

• **License: **GNU General Public License version 2

• **Any restrictions to use by non-academics: **None

## Abbreviations

API: Application Programming Interface; BHL: Biodiversity Heritage Library; DOI: Digital Object Identifier; ISSN: International Standard Serial Number; JSON: JavaScript Object Notation; OCR: Optical Character Recognition; URL: Uniform Resource Locator.

## Competing interests

The author declares that they have no competing interests.
